# Inositol monophosphatase 1 (IMPA1) promotes triple‐negative breast cancer progression through regulating mTOR pathway and EMT process

**DOI:** 10.1002/cam4.4970

**Published:** 2022-07-07

**Authors:** Shao‐Ying Yang, Yi‐Fan Xie, Tai‐Mei Zhang, Ling Deng, Li Liao, Shu‐Yuan Hu, Yin‐Ling Zhang, Fang‐Lin Zhang, Da‐Qiang Li

**Affiliations:** ^1^ Fudan University Shanghai Cancer Center and Institutes of Biomedical Sciences Fudan University Shanghai China; ^2^ Department of Breast Surgery, Shanghai Medical College Fudan University Shanghai China; ^3^ Cancer Institute, Shanghai Medical College Fudan University Shanghai China; ^4^ Shanghai Key Laboratory of Breast Cancer, Shanghai Medical College Fudan University Shanghai China; ^5^ Shanghai Key Laboratory of Radiation Oncology, Shanghai Medical College Fudan University Shanghai China

**Keywords:** IMPA1, metastasis, TNBC, tumor growth

## Abstract

Triple‐negative breast cancer (TNBC) is the most aggressive subtype of breast cancer, which is characterized by high heterogeneity and metabolic dysregulation. Inositol monophosphatase 1(IMPA1) is critical for the metabolism of inositol, which has profound effects on gene expression and other biological processes. Here, we report for the first time that IMPA1 was upregulated in TNBC cell lines and tissues, and enhanced cell colony formation and proliferation *in vitro* and tumorigenicity *in vivo*. Additionally, IMPA1 promoted cell motility *in vitro* and metastatic lung colonization *in vivo*. Mechanistic investigations by transcriptome sequencing revealed that 4782 genes were differentially expressed between cells with IMPA1 knockdown and control cells. Among the differentially expressed genes after IMPA1 knockdown, five significantly altered genes were verified via qRT‐PCR assays. Morerover, we found that the expression profile of those five targets as a gene set was significantly associated with IMPA1 status in TNBC cells. As this gene set was associated with mTOR pathway and epithelial‐mesenchymal transition (EMT) process, we further confirmed that IMPA1 induced mTOR activity and EMT process, which at least in part contributed to IMPA1‐induced TNBC progression. Collectively, our findings reveal a previously unrecognized role of IMPA1 in TNBC progression and identify IMPA1 as a potential target for TNBC therapy.

## Graphical abstract

IMPA1 was upregulated in TNBC tissues, and enhanced cell proliferation and metastatic potential. Five downstream targets of IMPA1 were identified, which are invovled in mTOR pahtway and EMT process to drive TNBC progression.

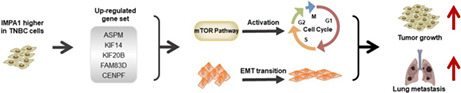

## 1 INTRODUCTION

Breast cancer is the most common malignancy worldwide and the second leading cause of cancer‐related death among women.^1^, ^2^  Triple‐negative breast cancer is characterized by the lack of estrogen receptor (ER), progesterone receptor (PR), and human epidermal growth factor receptor 2 (HER2),^3^ and accounts for 15%–20% of all breast cancers.^4^ TNBC is prone to early recurrence, metastasis, and poor prognosis than other types of breast cancer due to the lack of effective therapeutic targets.^5^ Therefore, the molecular mechanisms and new targets depending on the molecular profile of TNBC need to be explored.

In the last two decades, experimental and bioinformatics analysis of high‐throughput data generated from genomics^6^ and transcriptomics^7^ have been maturely applied in TNBC. Otherwise, as protein is the basic unit of cell biological process, proteomics analysis emerges as an essential tool to decipher the mechanisms of TNBC tumorigenesis and progression. By analyzing the proteomic data of 90 TNBC patients from our cancer center, we found inositol monophosphatase 1 (IMPA1) was upregulated in TNBC tissues compared to normal controls, but its functional role in TNBC progression has not been reported yet.

IMPA1 is required for dephosphorylating inositol monophosphates to generate inositol,^8^ which is an important metabolite as a precursor for generating phosphoinositide, and therefore has profound effects on gene expression and is critical for cell signaling and biological processes.^9^ Current studies have shown that dysregulation of the inositol cycle is associated with a variety of human diseases, including cancer, neurological diseases, and diabetes.^10^ It has been reported that IMPA1 mutation is associated with decreased brain functions, neuronal differentiation, and behavior disruption, and has been implicated as the pharmacological target of lithium action in brain.^11^, ^12^, ^13^ For example, IMPA1 mutation in intellectual disability patients impairs neurogenesis depending on the deregulation of gene expression in pathways necessary for neurogenesis.^13^ Recently, researchers have found that IMPA‐derived inositol modulates mitochondrial functions by targeting kinase AMPK.^14^ Additionally, another study reveals that high IMPA1 expression suggests a worse prognosis in diffuse large B‐cell lymphoma by integration of the differentially expressed miRNAs with matched mRNA profiles.^15^ However, IMPA1 expression profiles and its associated functional role in cancer progression, especially in TNBC, remain unexplored.

In this study, we report for the first time that IMPA1 was upregulated in TNBC cell lines and tissues, and increased cell proliferation and metastatic potential, and identified five downstream targets of IMPA1 which drive TNBC progression and metastasis through mTOR pathway and EMT process. Together, these findings suggest that IMPA1 might be a promising target for TNBC therapy.

## 2 MATERIALS AND METHODS

### 2.1 Cell culture and reagents

Human embryonic kidney 293 T (HEK293T), human mammary epithelial cell lines (HMEC and MCF10A), human breast cell lines (MDA‐MB‐231, MDA‐MB‐453, MDA‐MB‐468, MDA‐MB‐157, Hs578T, BT549, BT20, HCC1806, and HCC1937) cell lines were obtained from Shanghai Key Laboratory of Breast Cancer, Fudan University Shanghai Cancer Center. MDA‐MB‐231‐derived LM2‐4175 cells were obtained from Guohong Hu (University of Chinese Academy of Sciences), and SUM159PT cell line was kindly provided by Asterand. The above cell lines were authenticated by short tandem repeat profiling and monitoring cell vitality. MCF10A and HMEC cells were cultured in DMEM (BasalMedia, #L110) containing 5% donor horse serum (Gibco, #16050–114), 20 ng/ml epidermal growth factor (Sino Biological Inc, #10605 HNAE), 0.5 mg/ml hydrocortisone (Yeasen, #40109ES08), 10 mg/ml human recombinant insulin (Yeasen, #40107ES76), and 1% penicillin/streptomycin (BasalMedia, #S110B). The others were maintained in DMEM with 10% fetal bovine serum (Gibco, #10270–106), and 1% penicillin/streptomycin (BasalMedia, # S110B). Transwell chambers and Cell Counting Kit‐8 (CCK8) were purchased from Corning Falcon (#353097) and Yeasen (#40203ES92), respectively.

### 2.2 Tissue samples

Nine paired samples of TNBC tissues and adjacent control tissues were obtained from TNBC patients undergoing surgery at Fudan University Shanghai Cancer Center who did not receive radiotherapy, chemotherapy or endocrine therapy before surgery. After patients signed informed consent for sample collection, tissue samples were carefully stored in liquid nitrogen immediately after surgery. This experiment was approved by the ethics committee of Fudan University Shanghai Cancer Center. All procedures were performed in accordance with Ethical guidelines of the declaration of Helsinki.

### 2.3 Dataset

Expression profiles of The Cancer Genome Atlas (TCGA) cohorts and the Molecular Taxonomy of Breast Cancer International Consortium (METABRIC) cohorts were downloaded from website (https://portal.gdc.cancer.gov/) and (http://www.cbioportal.org/), respectively. GSE76275 dataset deposited in National Center for Biotechnology Information (NCBI) Gene Expression Omnibus (GEO) database was downloaded from website (https://www.ncbi.nlm.nih.gov/geo/). RNA‐seq data (tumor tissues: *n* = 360; paired normal tissues: *n* = 88), and protein proteomic data (tumor tissues: *n* = 90; paired normal tissues: *n* = 72, proteomic data not published yet) of the present study were obtained from patients in the Fudan University Shanghai Cancer Center (FUSCC) TNBC Project (FUSCC cohort). The web interface tool named ‘GSCA’ was used to retrieve Breast cancer mRNA expression data of input genes.^16^

### 2.4 Expression vectors

IMPA1 cDNA was amplified by PCR and subcloned into lentiviral vector pLVX‐IRES‐NEO (Biofeng, #632181) to generate pLVX‐HA‐IMPA1 construct. IMPA1 short hairpin RNA (shRNA) primers were obtained from http://rnaidesigner.thermofisher.com/, synthesized from HuaGene Biotech, then subcloned into pLKO.1‐TRC vector (Addgene, #10878). The DNA constructs used in molecular cloning were listed in Table S1. Primers used in molecular cloning were listed in Table S2. The detailed information of shIMAP1 target sequences was provided in Table S3. All constructs were sequenced correctly before using.

### 2.5 Plasmid, shRNA transfection, and viral transduction

Plasmid and shRNA transfection were carried out in HEK293T cells during logarithmic growth phase. Plasmids of target genes and packaging vectors as well as DNA transfection reagent (TENGYI, #TF201201) were mixed in 300 μl Opti‐MEM Reduced Serum Medium (ThermoFisher, #31985070). After 15 to 20 min, the mixture was carefully delivered to HEK293T cells according to the manufacturer's protocol. After 48 h of transfection, the virus was collected, filtered, and aliquoted in 1.5 ml sterile EP tube and frozen at −80°C. To generate cell lines expressing shIMPA1 or HA‐IMPA1, cells were plated in 6‐mm dishes and infected with shIMPA1 or HA‐IMPA1 viruses supplemented with 8 μg/ml of polybrene (Sigma‐Aldrich, #H9268). After 48 h of infection, 2 μg/ml puromycin (Sangon, #A610593‐0025) was used to select cells with stable expressing shIMPA1, and 500 μg/ml G418 (Sangon, #A600958‐0005) was used to select cells with stable expressing HA‐IMPA1.

### 2.6 RNA extraction, RT‐PCR, and qPCR

RNA from cell pellets was extracted with RNAiso Plus (Takara, #9109), and the concentration was measured. Subsequently, 1 μg RNA was used for reverse transcription to obtain cDNA using PrimeScript RT Master Mix (Takara, #RR036A) according to the manufacturer's protocol. The obtained cDNA was diluted 10‐fold and used for qPCR experiments with TB Green Premix Ex Taq (Takara, #RR420A). The 2 ^−∆∆ CT^ method was used to calculate the relative mRNA expression levels, and the qPCR primers were synthesized in GENEWIZ and primers sequences were provided in Table S4.

### 2.7 Antibodies and Western blot

Tissues and cells were lysed with RIPA buffer containing protease inhibitor (Bimake, #B14002) and phosphatase inhibitor (Bimake, #B15003). Bicinchoninic acid (BCA) protein assay kit (Yeasen, #20201ES90) was used to detect protein concentration. Equal quantity of proteins was separated by SDS‐PAGE and transferred to PVDF membrane. The membrane was blocked with 5% bovine serum albumin (Yeasen, #36101ES80) for 1–3 h at room temperature, and subsequently incubated with primary antibodies overnight at 4°C, followed by incubation with secondary antibodies for 1–3 h at room temperature. Next, the electrochemiluminescence reagents (Yeasen, #36208ES76) were used to detect the antibody signals, and quantitative analysis of the developed results was performed by ImageJ software using vinculin as an internal reference to calculate the relative expression levels of target protein. Primary antibodies of IMPA1(Abcam, #ab184165), Vinculin (Sigma, #V9131), and HA (CST, #3724S) were used in this study. Other antibody information is shown in Table S5.

### 2.8 Cell proliferation and colony formation assays

Cells were seeded in 96‐well plates at a density of 1000 cells in 100 μl DMEM per well and three replicate wells were included in each group. After incubation at 37°C in an environment with 5% CO_2_ for 24, 48, 72 and 96 h, 10 μl CCK8 (Yeasen, #40203ES88) solution was added to each well and reacted for 2 h, the absorbance values at 450 nm were monitored. For colony formation assays, cells were seeded in 6‐well plates at a density of 1000 cells in 2 ml DMEM per well in triplicates for each group, and the cells were harvested after 14 days. Colonies were fixed with methanol for 30 min and stained with 0.5% crystal violet for 30 min. Total number of colonies in each well was counted.

### 2.9 Cell‐cycle analysis

Cells cultured for 48 h were digested with trypsin–EDTA, and 1 × 10^5^–1 × 10^6^ cells were harvested after resuspension with PBS and fixed with 70% ethanol overnight. After washing with PBS, propidium staining was added, followed by 30 min reaction at 37°C protected from light, the samples were sent for flow cytometric analysis.

### 2.10 Cell migration and wound‐healing assays

For observing cell migration ability, cells were digested using 0.25% trypsin for 3 min, single‐cell suspension was prepared, and cell number was counted in FBS free medium. And 4 × 10^4^ cells in 200 μl FBS free medium were placed in the upper chamber of transwell chamber, and 800 μl medium containing 5% FBS were added to the lower chamber of well. After 20 h of normal culture, the cells in the lower surface of chambers were fixed for 30 min using methanol, stained with 0.5% crystal violet for 30 min, and photographed under a microscope and counted eight random fields under a microscope. For wound‐healing assays, cells were cultured in a 6‐well plate, and 200 μl pipette tips were used to scratch. Then cells were washed with PBS for three times and taken photos under a microscope. Next, cells were cultured in FBS free medium for 24 h, and photos were taken under a microscope to observe the wound healing rate.

### 2.11 Cell morphology photography

Cell morphology of control and IMPA1‐depleted LM2‐4175 and MDA‐MB‐231 cells was observed under a microscope.

### 2.12 Immunofluorescent staining

A total of 8 × 10^4^ cells were cultured on cell climbing slices. After 24 h, cells were washed three times with PBS, fixed at room temperature for 30 min with 4% paraformaldehyde, and then blocked with 10% normal goat serum in PBST for 1 h. The corresponding antibodies were incubated overnight. After cells were washed for three times using PBST, the secondary antibodies conjugated with Alexa Fluor488 (CST, #4408S or #4412S) or Alexa Fluor 555 (CST, #4409S or #4413S) were added at room temperature without light for 30 min. DNA was stained with the fluoroshield mounting medium with DAPI (Abcam, #ab104139).

### 2.13 Tumor xenografts, lung metastasis in nude mice

The laboratory animal protocols were approved by the Institutional Animal Care and Use Committee of Shanghai Cancer Center, Fudan University, and the ethics and care of animals were conducted following institutional guidelines. For subcutaneous inoculation, 1 × 10^6^ LM2‐4175 cells stably expressing shNC or shIMPA1 were inoculated subcutaneously in 5‐week‐old BALB/c female nude mice (Shanghai Cancer Center, Shanghai, China). The tumor growth kinetics were measured three times a week and calculated by the formula (length × width^2^)/2. The mice were sacrificed 4 weeks later using carbon dioxide asphyxiation, and the removed tumors were weighed and calculated. For experimental lung metastasis assays, 0.5 × 10^6^ LM2‐4175 cells stably expressing shNC or shIMPA1 were inoculated into tail veins of 5‐week‐old BALB/c female nude mice. Five weeks later, the mice were sacrificed, and lungs were dissected and metastatic nodules were observed under microscope. H&E staining was carried out to examine the presence of micrometastases in paraffin‐embedded lung tissues.

### 2.14 Statistical analysis

All experiments were set up for at least three replicates, and data are presented with mean ± standard deviation. Alteration significance between two groups was calculated using unpaired two‐tailed Student's *t*‐test in GraphPad prism 8, and *p‐*value <0.05 was considered statistically significant.

## 3 RESULTS

### 3.1 IMPA1 is upregulated in TNBC tissues

**FIGURE 1:**
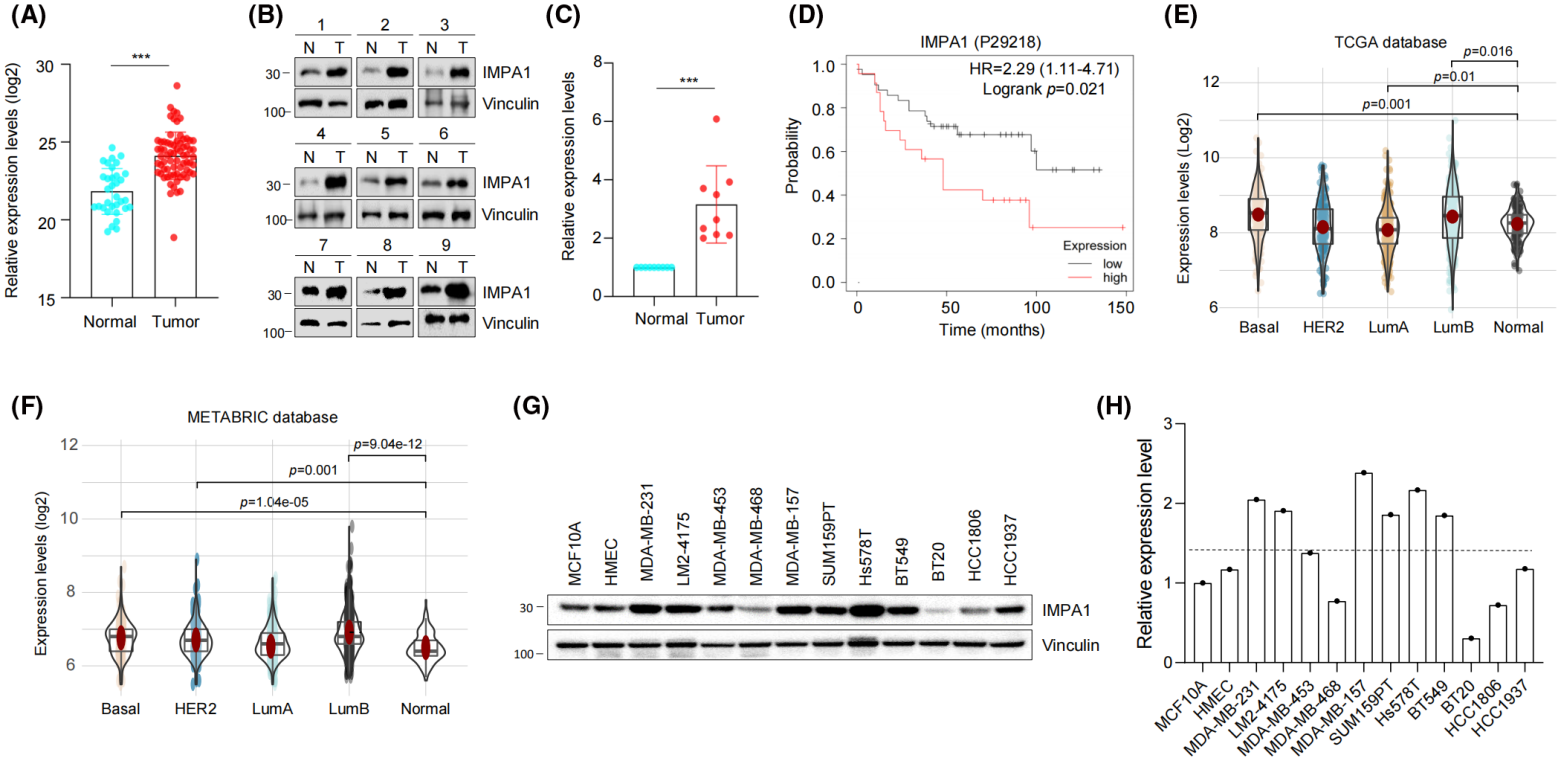
IMPA1 is upregulated in TNBC tissues. (A) Analysis of IMPA1 protein levels in 90 primary TNBC tissues and 72 adjacent normal tissues using proteomic data in FUSCC cohort. (B, C) Western blotting analysis of IMPA1 expression levels in 9 pairs of TNBC and matched normal breast tissues using Vinculin as a loading control (B) and quantitative results (C) are shown. (D) Survival analyses of IMPA1 protein in breast cancer (OS in Kaplan–Meier Plotter). *p* < 0.05 was considered statistically significant. (E, F) Analysis of IMPA1 mRNA levels across differential breast cancer subtypes in the TCGA database (E) and METABRIC database (F). (G, H) Western blot was used to detect the expression levels of IMPA1 in eleven TNBC cell lines and two human mammary epithelial cell lines (G) and quantitative results (H) are shown.

A proteomic analysis has been carried out to screen differentially expressed proteins in 90 TNBC samples (data unpublished yet), and we re‐analyzed these data and found that the expression levels of IMPA1 were significantly higher in TNBC tissues than normal counterparts (Figure 1A). To further confirm the expression pattern of IMPA1 in TNBC, we additionally collected nine pairs of TNBC and its paired control samples, and examined the protein levels of IMPA1 by Western blot. Results showed that IMPA1 protein levels were significantly higher in TNBC tissues as compared with their normal counterparts (Figure 1B,C). Due to the lack of clinical information of IMPA1‐expressing TNBC samples, we analyzed a previously published proteomic dataset of breast cancer documented in KMplotter database (www.kmplot.com). As shown in Figure 1D, high IMPA1 status was significantly associated with a worse breast cancer prognosis.

As protein abundance variation may be modulated by its mRNA level, copy number variation (CNV), gene mutations, DNA methylations, and so on,^17^ we then analyzed the mRNA levels of IMPA1 in breast cancer from The Cancer Genome Atlas (TCGA) and the Molecular Taxonomy of Breast Cancer International Consortium (METABRIC) cohorts. It could be found that the mRNA levels of IMPA1 in TNBC subtype were significantly higher than that in normal tissues (Figure 1E,F). Moreover, an integrated database named Gene Set Cancer Analysis (GSCA) that was used for genomic gene set cancer analysis was adopted for further analyzing and obtaining more genomic information about IMPA1 in breast cancer.^16^, ^17^ Results showed that CNV of IMPA1 was significantly higher in breast cancer and was closely related to its mRNA level, but few mutations were identified in IMPA1 (Figure S1A–C). In addition, although methylation of IMPA1 was associated with its mRNA level, it was not significantly altered in breast cancer compared with normal control (Figure S1D). Furthermore, we examined IMPA1 levels in 2 normal breast epithelial and 11 representatives TNBC cell lines by immunoblotting (Figure 1G). As shown in Figure 1H, IMPA1 was upregulated in 8 of 11 (72.7%) TNBC cell lines as compared with two normal counterparts. These results suggest that IMPA1 is upregulated in TNBC cell lines and tissues, and high expression of IMPA1 is associated with the survival of breast cancer patients.

### 3.2 IMPA1 promotes TNBC proliferation and colony formation *in vitro*

**FIGURE 2:**
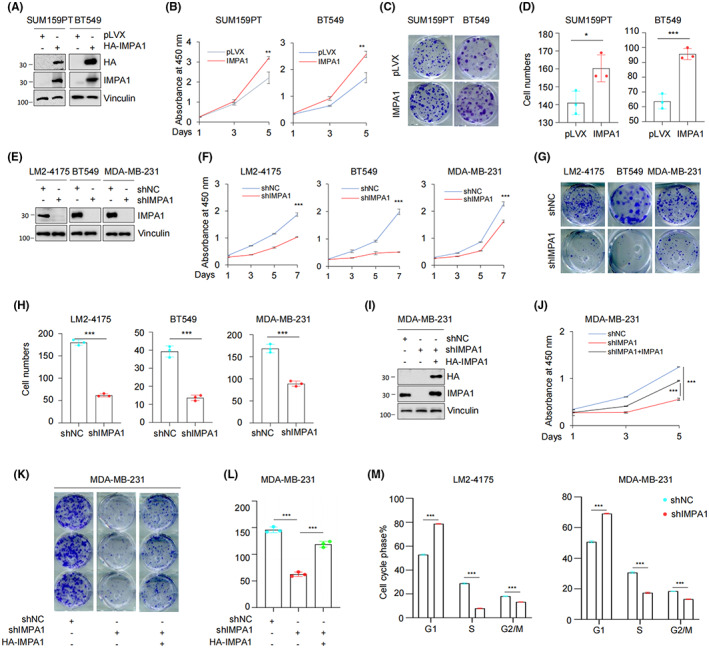
IMPA1 promotes TNBC cells proliferation, colony formation *in vitro*. (A) SUM159PT and BT549 cells stably expressing pLVX and HA‐IMPA1 were verified using immunoblotting with the corresponding antibodies. (B–D) SUM159PT and BT549 cells stably expressing pLVX and HA‐IMPA1 were subjected to cell proliferation assays using CCK‐8 kit (B), and colony formation assays (C), and the corresponding quantitative results (D) are shown. (E) LM2‐4175, BT549, and MDA‐MB‐231 cells stably expressing shNC and shIMPA1 were verified using immunoblotting with the corresponding antibodies. (F–H) LM2‐4175, BT549, and MDA‐MB‐231 cells stably expressing shNC and shIMPA1 were used to investigate cell proliferation by CCK‐8 assays (F) and colony formation assays (G) and the corresponding quantitative results (H) are shown. (I) Verification of reverted IMPA1 expression after knockdown in MDA‐MB‐231 cells. (J–L) MDA‐MB‐231 cells with reverted IMPA1 expression were used to detect cell proliferation by CCK‐8 assays (J) and colony formation assays (K) and the corresponding quantitative results (L) are shown. (M) Cell‐cycle distribution was analyzed by flow cytometric assays in LM2‐4175 and MDA‐MB‐231 cells stably expressing shNC and shIMPA1 and the corresponding quantitative results are shown. *p* < 0.05 was considered statistically significant.

To investigate the biological function of IMPA1 in TNBC, we generated stable SUM159PT and BT549 cell lines overexpressing HA‐IMPA1 by infection of lentiviral vectors. As shown in Figure 2A and Figure S2A, HA‐IMPA1 had been successfully overexpressed in SUM159PT and BT549 which were verified at both protein and mRNA levels. CCK8 cell proliferation and colony formation assays suggested that, compared to empty vector controls, overexpression of IMPA1 accelerated cell proliferation (Figure 2B) and colony formation (Figure 2C,D) of SUM159PT and BT549 cells. In contrast, knockdown of endogenous IMPA1 in LM2‐4175, MDA‐MB‐231, and BT549 cells using shIMPA1 lentiviral vectors (Figure 2E and Figure S2B), significantly decreased cell proliferation (Figure 2F) and colony formation capability (Figure 2G,H). To further confirm the pro‐proliferative effect of IMPA1, we reverted the expression of IMPA1 in MDA‐MB‐231 cells in which IMPA1 was knocked down (Figure 2I). It was found that the inhibited proliferation caused by IMPA1 knockdown could be partially rescued (Figure 2J–L). Flow cytometric assays suggested that knockdown of IMPA1 remarkably decreased cell percentage in S and G2/M phase (Figure 2M and Figure S2C,D)). All the above results suggest that IMPA1 promotes the proliferate potential and clonogenicity of TNBC cells *in vitro*.

### 3.3 IMPA1 enhances TNBC cell migration *in vitro*

**FIGURE 3:**
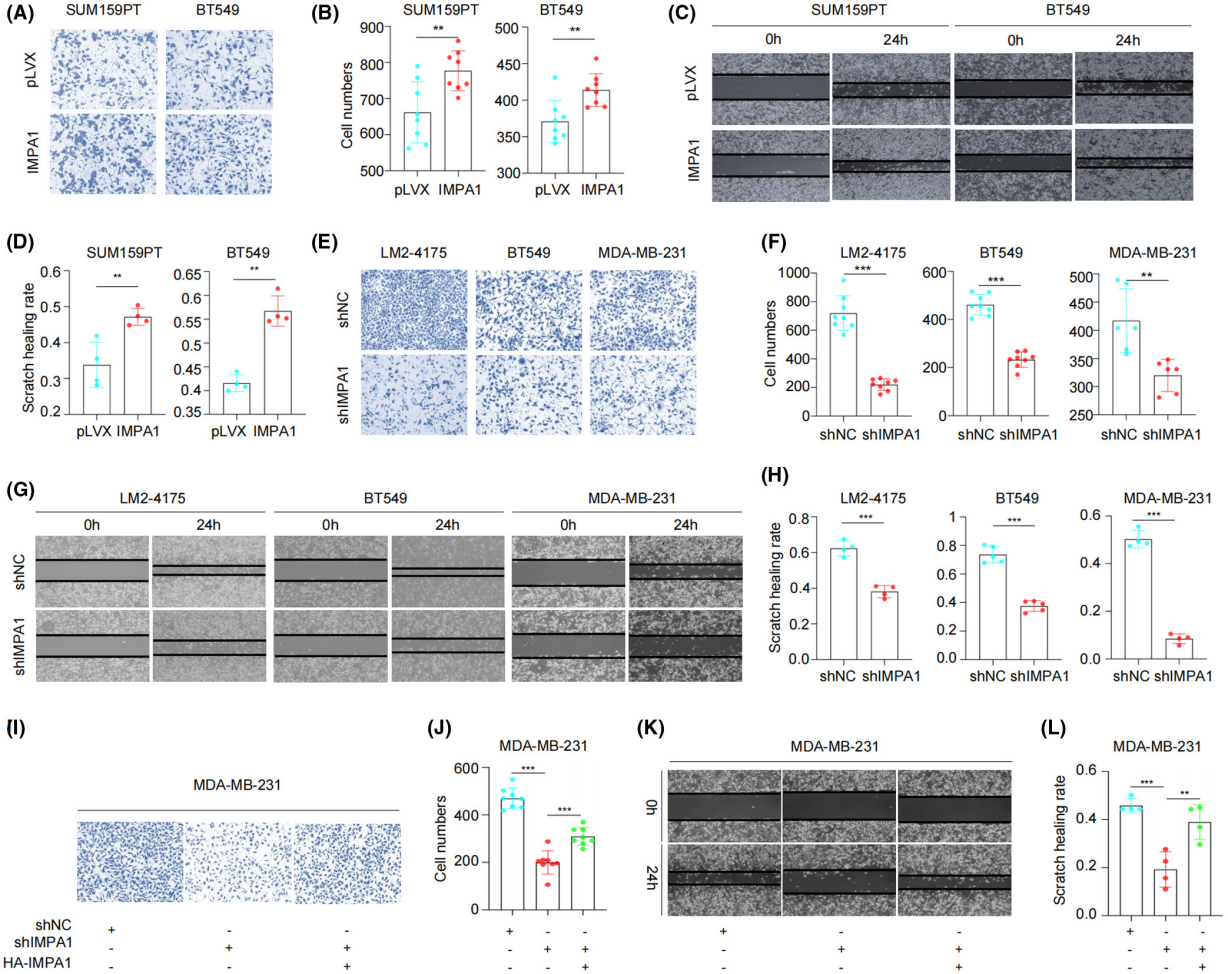
IMPA1 enhances TNBC cell migration potential *in vitro*. (A, B) SUM159PT and BT549 cells stably expressing pLVX and HA‐IMPA1 were used to detect cell migration ability by Transwell assays (A) and the corresponding quantitative results (B) are shown. (C, D) SUM159PT and BT549 cells stably expressing pLVX and HA‐IMPA1 were used to detect cell mobility by wound‐healing assays (C) and the corresponding quantitative results (D) are shown. (E, F) LM2‐4175, BT549, and MDA‐MB‐231 cells stably expressing shNC and shIMPA1 were used to detect cell migration ability by Transwell assays (E) and the corresponding quantitative results (F) are shown. (G, H) LM2‐4175, BT549, and MDA‐MB‐231 cells stably expressing shNC and shIMPA1 were used to detect cell mobility by wound‐healing assay (G) and the corresponding quantitative results (H) are shown. (I, J) MDA‐MB‐231 cells with reverted IMPA1 expression were used to detect cell migration ability by Transwell assays (I) and the corresponding quantitative results (J) are shown. (K, L) MDA‐MB‐231 cells with reverted IMPA1 expression were used to detect cell mobility by wound‐healing assays (K) and the corresponding quantitative results (L) are shown. *p* < 0.05 was considered statistically significant.

High migratory and metastatic ability is an important pathologic characteristic of TNBC cells. Therefore, we next examined the effect of IMPA1 on the migration potential of TNBC cells. Transwell and Wound‐healing assays revealed that overexpression of IMPA1 in SUM159PT and BT549 cells significantly increased migration potential as compared with control cells (Figure 3A–D). In contrast, compared to control cells, shIMPA1‐infected LM2‐4175, BT549, and MDA‐MB‐231 cells showed a significant decrease in migratory phenotype (Figure 3E–H). More importantly, the inhibited migration caused by IMPA1 knockdown could be partially rescued by re‐expressing IMPA1 (Figure 3I–L). Together, these results indicate that IMPA1 strengthens migration potential of TNBC cells *in vitro*.

### 3.4 IMPA1 promotes xenograft tumor growth and metastasis *in vivo*

**FIGURE 4:**
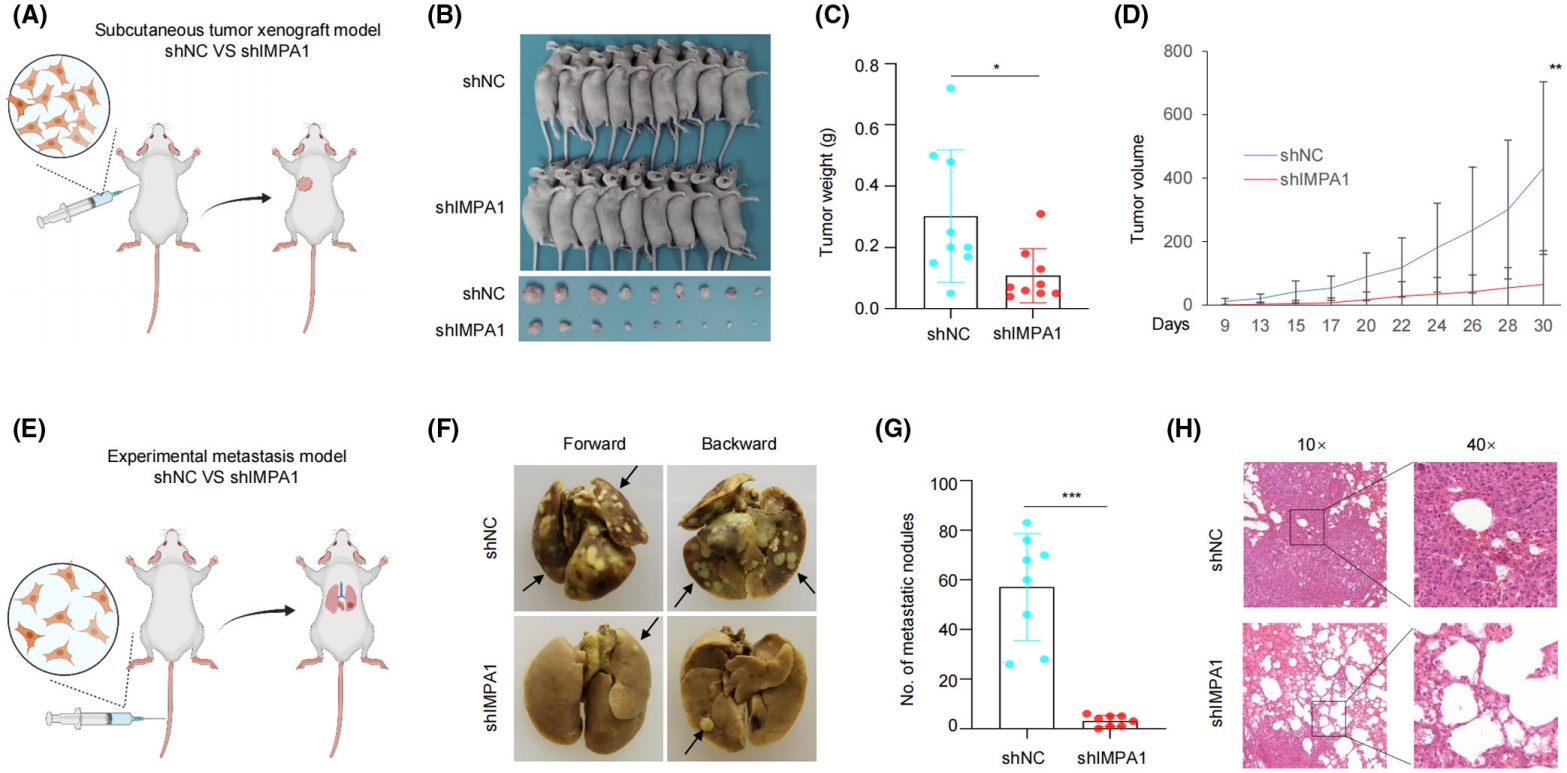
IMPA1 promotes TNBC xenograft tumor growth and metastasis *in vivo*. (A) Schematic presentation of the subcutaneous tumor xenograft model of LM2‐4175 cells stably expressing shNC and shIMPA1. A total of 1 × 10^6^ cells per mouse, *n* = 9 mice per group. Drawing by BioRender.com. (B, C) Images of mice and tumors (B) as well as tumor weight (C) after 4 weeks of tumor cells injection  are shown. (D) Primary tumor growth rate in each experimental groups in (B) measured three times per week. (E) Schematic presentation of the experimental lung metastasis model. LM2‐4175 cells stably expressing shNC and shIMPA1 were injected intravenously into BALB/c female nude mice to develop experimental lung metastasis. A total of 0.5 × 10^6^ cells per mouse, *n* = 8 mice per group. Drawing by BioRender.com. (F–H) Representative images of lungs (F) and quantification of metastatic nodules (G) at 5 weeks after injection, and H&E staining of metastatic lesions in lung (H) are shown.

In order to further verify the results of *in vitro* experiments, LM2‐4175 cells stably expressing shIMPA1 or shNC were inoculated subcutaneously into 5‐week‐old female BALB/c nude mice. As shown in Figure 4A–D, the tumor growth rate in shIMPA1 group was significantly slower than that in shNC group (Figure 4D), and the tumor weight and volume were significantly lower than those in the shNC group (Figure 4C). These data indicate that IMPA1 promotes tumorigenic capacity of TNBC cells *in vivo*. Additionally, to address whether IMPA1 affects TNBC metastasis *in vivo*, LM2‐4175 cells stably expressing shIMPA1 or shNC were injected into the tail vein of 5‐week‐old female BALB/c nude mice. As shown in Figure 4E–G, IMPA1 knockdown decreased pulmonary metastatic nodules in the nude mice as compared with those of control group. These results were also confirmed by H&E staining of the dissected lung sections (Figure 4H). In summary, these results suggest that IMPA1 knockdown reduces TNBC cell tumorigenic potential and lung metastatic capacity *in vivo*.

### 3.5 Analysis of molecular pathways regulated by IMPA1 using transcriptome sequencing

**FIGURE 5:**
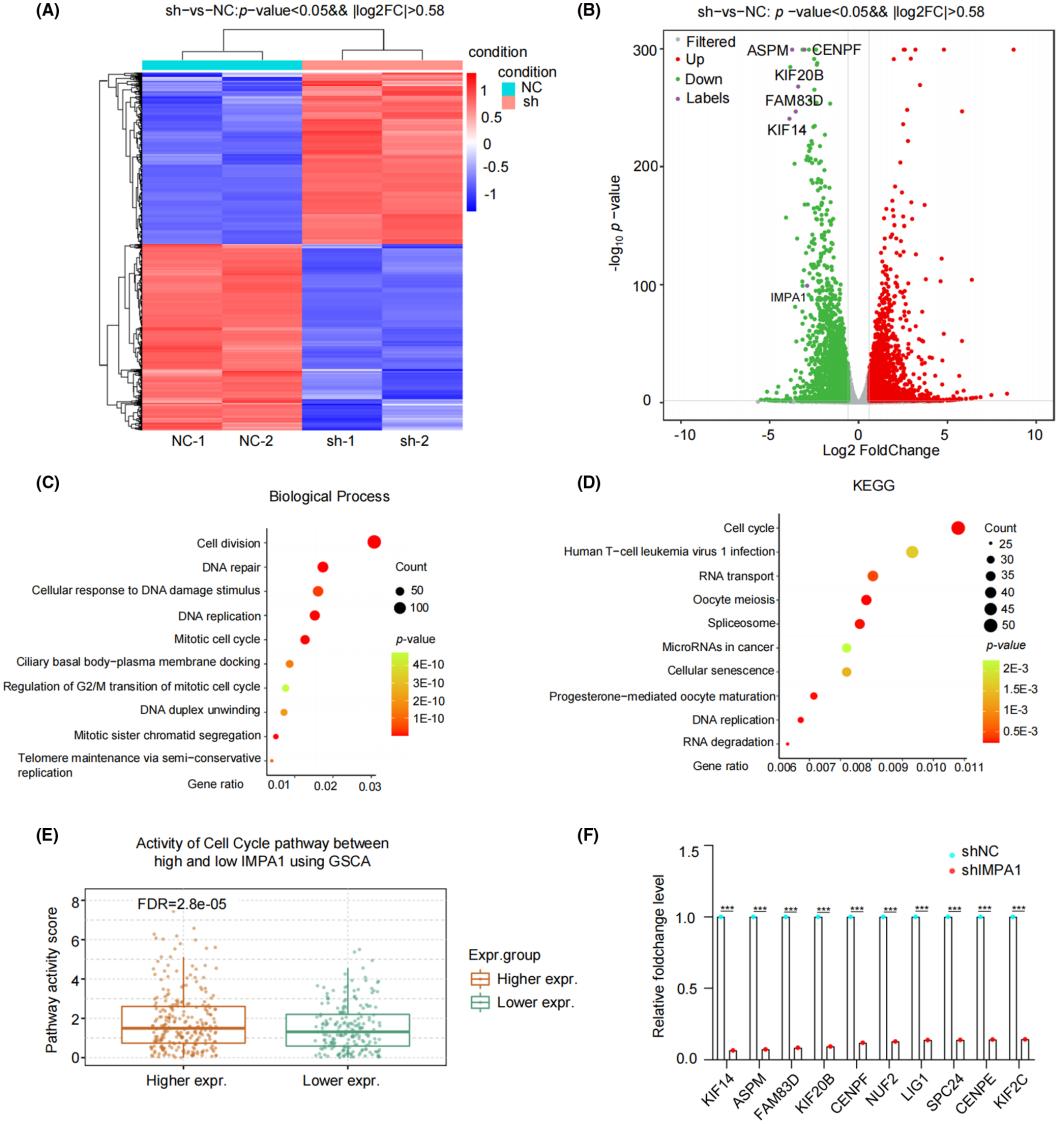
Analysis of molecular pathways regulated by IMPA1 using transcriptome sequencing. (A, B) Heatmap and volcano plot showed differentially expressed genes in cells with IMPA1 knockdown as compared with control cells. (C) GO analysis of down‐regulated target genes by IMPA1 knockdown according to biological process. (D) Bubble chart of KEGG analysis of down‐regulated target genes by IMPA1 knockdown. (E) Activity of cell‐cycle pathway analyzed using GSCA online tool between high and low IMPA1 groups. (F) The potential target genes associated with cell division significantly altered by IMPA1 knockdown revealed by transcriptome sequencing.

To explore how IMPA1 exerted biological functions in TNBC cells, transcriptome sequencing was performed. We transfected shIMPA1 or shNC into LM2‐4175 cells to establish stable cell lines, and then carried out RNA‐seq to define potential target genes regulated by IMPA1. Differential expression profile of LM2‐4175 cells after IMPA1 knockdown is shown in heatmap (Figure 5A). As shown in Figure 5B, using a cutoff of *p*‐value <0.05 and fold change >1.5, we identified 4782 differentially expressed mRNAs in IMPA1‐knocked down cells (downregulated genes: 2486; upregulated genes: 2296). We subsequently subjected the alternated genes to Gene Ontology (GO) and Comparative Kyoto Encyclopedia of Genes and Genomes (KEGG) analysis. GO analysis revealed that the downregulated genes induced by IMPA1 knockdown were classified into three ontologies, including biological process (BP), molecular function (MF) and cellular component (CC) (Figure 5C and Figure S3A,B). Cell division (GO:0051301), DNA replication (GO:0006260), mitotic cell cycle (GO:0000278) and other top 10 GO‐BP annotation terms are shown in Figure 5C. Additionally, KEGG functional annotations revealed that cellcycle pathways were significantly changed in the response to IMPA1 knockdown (Figure 5D).

Furthermore, we downloaded three TNBC cohorts from TCGA, METABRIC, and GSE76275 dataset, and divided them into IMAP1 high and low groups, respectively. Through Gene set enrichment analysis (GSEA), we found that cell‐cycle signature was significantly enriched in IMAP1‐high group (Figure S3C–E). In addition, the GSCA database analysis result was consistent with Figure S3C–E, which showed that cell‐cycle activity was significantly elevated in IMAP1‐high breast cancer group (Figure 5E). As shown in Figure 5F, the downregulated top 10 cell division associated genes are listed according to their relative fold‐change. These results demonstrated that IMPA1 exhibits oncoprotein potential dependent, at least in part, on cell proliferation associated pathways.

### 3.6 IMPA1 exerts functional role partly through its downstream five targets

**FIGURE 6:**
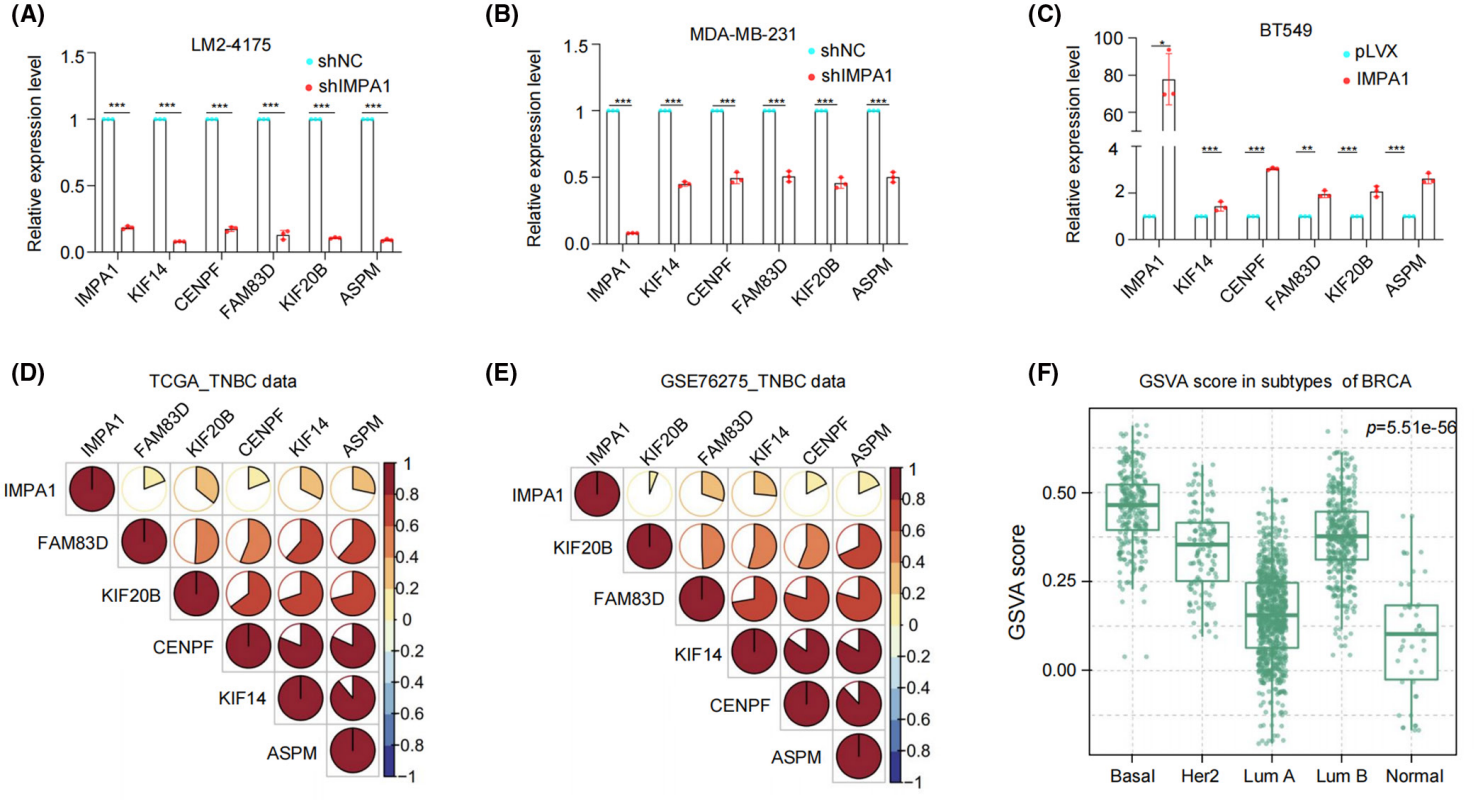
Validation of five target genes regulated by IMPA1. (A–C) Verification of representative five target genes of KIF14, CENPF, FAM83D, KIF20B, and ASPM by qRT‐PCR in cells in which IMPA1 was knocked down (A, B) or overexpressed (C). (D, E) Correlation analysis of the gene set in TNBC cohorts deposited in TCGA (D) and GSE76275 dataset of GEO (E) databases. (F) The gene set including five target genes and IMPA1 was used for gene set variable analysis (GSVA) to investigate genomic variations in different subtypes of breast cancer.

The top five genes showed in Figure 5F including the Kinesin Family Member 14 (KIF14), Assembly Factor for Spindle Microtubules (ASPM), Family with Sequence Similarity 83 Member D (FAM83D), Kinesin Family Member 20B (KIF20B), and Centromere Protein F (CENPF) were selected to verify RNA‐seq analysis results by qRT‐PCR. Results showed that knockdown of IMPA1 significantly reduced the mRNA levels of those five target genes (Figure 6A,B), whereas overexpression of IMPA1 upregulated the expression levels of 5 target genes (Figure 6C). Moreover, we confirmed that the mRNA levels of five targets were upregulated in TNBC compared to in adjacent tissues using RNA‐sequencing data from FUSCC cohort (Figure S4A), and were positively correlated with IMPA1 status (Figure S4B–F). Besides, Correlation analysis using TNBC cohorts deposited in TCGA (Figure 6D), GSE76275 dataset (Figure 6E) and METABRIC databases (Figure S4G) showed the correlation of expression levels between IMAP1 and those five targets. Therefore, we used those five target genes and IMPA1 as a gene set to investigate genomic variations by gene set variable analysis (GSVA) of breast cancer, and found that it was significantly higher in breast cancer (Figure S4H), especially in TNBC (Figure 6F), and significantly correlated with cell‐cycle pathway activity (Figure S4I). Taken together, we identify five targets of IMPA1 which function as downstream effectors of IMPA1.

### 3.7 IMPA1 exhibits oncogenic role through mTOR pathway and EMT process

Deciphering signaling pathway networks is important for understanding the molecular mechanisms of IMPA function. As previously reported, KIF14 promotes the proliferation by regulating the phosphorylation of AKT,^18^ and ASPM regulates lung adenocarcinoma cell metastasis by promoting epithelial‐mesenchymal transition (EMT) through the PI3K/Akt signaling pathway.^19^ FAM83D is associated with EMT process^20^ and regulates cancer cell proliferation and migration through Akt/mTOR pathway.^21^ Moreover, KIF20B mediates colorectal cancer cell migration and invasion though promoting EMT process.^22^ CENPF regulates bone metastasis of breast cancer through its ability to activate PI3K‐AKT‐mTORC1.^23^ Therefore, we hypothesis that IMPA1 promotes cell proliferation and migration through Akt/mTOR/p70S6k and EMT process. Next, we subjected the five target genes as a gene set to GSCA analysis to analyze the mTOR pathway variations, and found that it was significantly correlated with TSC‐mTOR pathway in breast cancer (Figure S5A). And TSC‐mTOR pathway activity was significantly elevated in IMAP1‐high breast cancer group as shown in Figure S5B.

**FIGURE 7:**
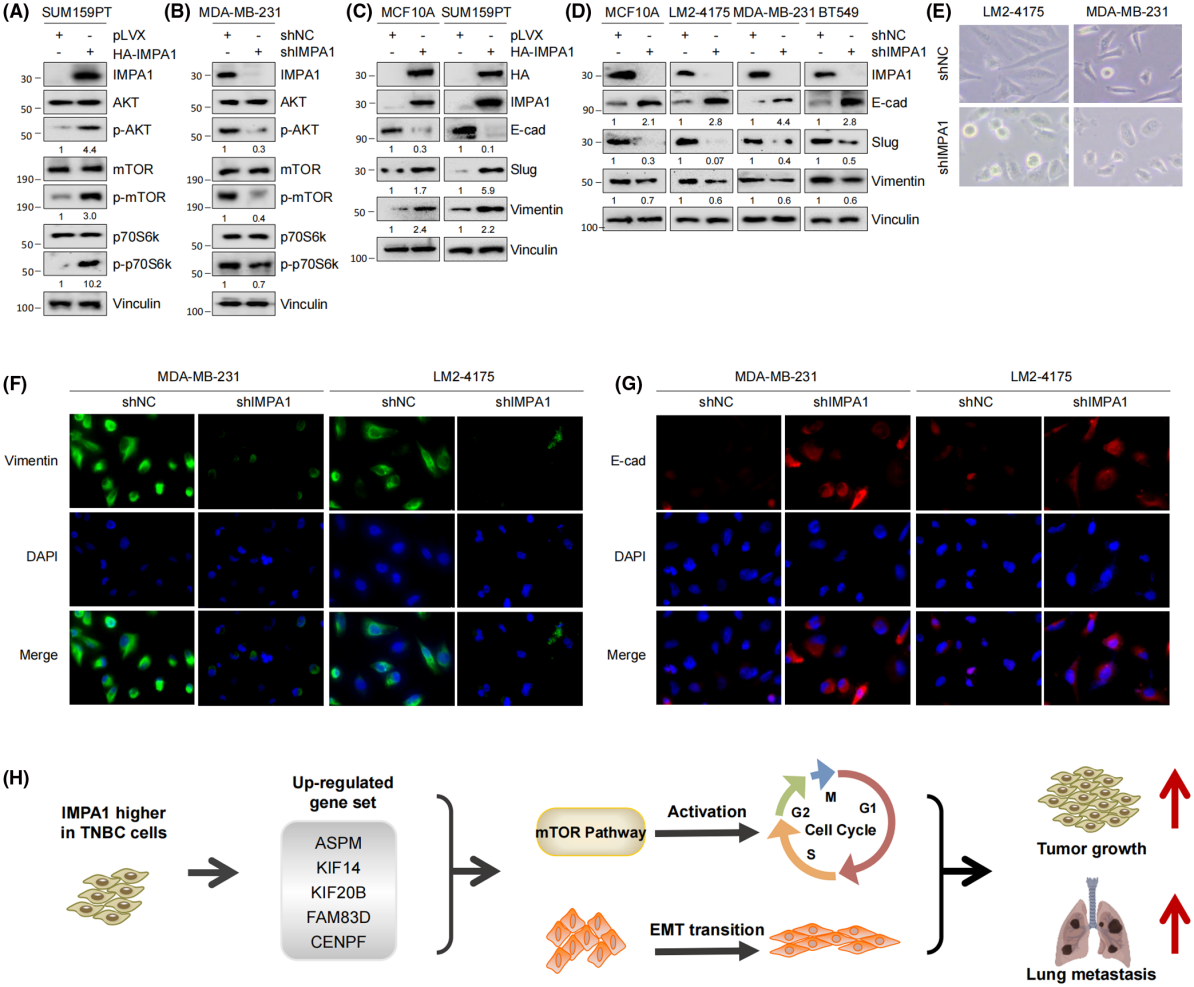
IMPA1 exhibits oncogenic roles through mTOR pathway and EMT process. (A, B) Immunoblotting was used to detect the levels of Akt/mTOR/p70S6k and their phosphorylation in cells with IMPA1 overexpression (A) and knockdown (B), respectively. (C, D) The expression levles of EMT related makers were detected by immunoblotting in cells with IMPA1 overexpression (C) and knockdown (D), respectively. (E) Representative cell morphology of MDA‐MB‐231 and LM2‐4175 cells stably expressing shNC and shIMPA1. (F, G) MDA‐MB‐231 and LM2‐4175 cells stably expressing shNC and shIMPA1 were subjected to immunofluorescent staining with indicated antibodies. (H) The proposed working model.

Through immunoblotting analysis, we found that the phosphorylation levels of Akt, mTOR, and p70S6k were all significantly increased in cells stably expressing HA‐IMPA1 compared with control cells (Figure 7A). Whereas the phosphorylation levels of the above molecules showed a decrease in IMPA1knocked down cells (Figure 7B). In addition, we examined the expression levels of EMT associated molecules E‐cadherin, Slug, and Vimentin in IMPA1 overexpressing and knockdown cells, respectively. As shown in Figure 7C, expression of the epithelial marker E‐cadherin was reduced, and mesenchymal markers Slug and Vimentin were increased in cells overexpressing IMPA1. Conversely, cells with IMPA1 knockdown increased in E‐cadherin expression and decreased in Slug and Vimentin expression compared with control cells (Figure 7D). Cell morphology of MDA‐MB‐231 and LM2‐4175 cells stably expressing shIMPA1 showed reduced mesenchymal characteristics (Figure 7E). In addition, the immunofluorescence assay results in Figure 7F and G showed consistent conclusion. Collectively, these data indicate that IMPA1 promotes TNBC proliferation and metastasis at least partly through five downstream targets and its downstream signaling pathways of mTOR pathway and EMT process.

## 4 DISCUSSION

In this study, we revealed for the first time that IMPA1 was upregulated in TNBC cell lines and tumor tissues, and exerted an oncogenic role in TNBC growth and metastasis through upregulating five downstream targets and their associated mTOR pathway and EMT process.

Proteins are the functional executors in cells. Although genomic and transcriptomic analysis are widely used in cancer research, global proteomic analysis of cancer that directly measures such changes is desirable. TNBC is a highly heterogeneous disease,^24^ and clinical evidence suggests that the cause of death in most patients is distant metastasis.^25^ Therefore, it is important to understand the molecular mechanisms of protumorgenic and metastatic process. By reanalyzing the quantitative proteomic data of TNBC samples in Shanghai cancer center, we found IMPA1 was upregulated in TNBC tumor tissues (Figure 1). Although many previous reports imply the association between IMPA1 and brain disfunctions,^11^, ^13^ there is no study of IMPA1 as tumor‐associated promotor. Here, we found that IMPA1 increased TNBC cell proliferation and migration capacity (Figures 2 and 3), and promoted xenograft tumor growth and metastasis (Figure 4). Therefore, IMPA1 exerts an oncogenic role in TNBC growth and metastasis. IMPA2 is an important paralog of IMPA1 and plays an oncogenic role in cervical cancer.^26^ Whether the functions between IMPA1 and IMPA2 are complementary remains to be explored. According to The Human Protein Atlas database, IMPA2 can not be detected in breast tissues, while IMPA1 is present at moderate levels. In addition to the different expression patterns, the activity of IMPA2 on inositol monophosphate is significantly lower than that of IMPA1.^27^ Therefore, it is IMPA1 that plays an important role in TNBC progression.

To address the mechanism by which IMPA1 exerts biological functions and increases TNBC progression, RNA‐seq analysis was performed on IMPA1‐knocked down LM2‐4175 cells. Results showed that IMPA1 promoted TNBC progression partially through affecting cell‐cycle associated genes (Figure 5 and Figure S3). We then selected five significantly changed targets, and confirmed that expression of these targets was upregulated in TNBC. In addition, these target genes were detected positively related to IMPA1 expression status in RNA‐sequencing data in cohorts from FUSCC, TCGA, METABRIC, and GSE76275 dataset, and verified by qPCR in TNBC cell lines (Figure 6 and Figure S4). In agreement, the gene set including those five targets and IMPA1 was significantly enriched in cell cycle by GSEA analysis. These genes have been reported closely related to cell cycle signaling which may account for the IMPA1‐induce cell proliferation.^28^, ^29^, ^30^ Although IMPA1 has profound effects on gene expression, it is not a transcription factor which plays direct role on gene transcription regulation. Thus, it is important to figure out how IMPA1 controls the expression of cell cycle‐related genes. It has been reported that aberration in IMPA1 enzyme activity causes inositol metabolism disorders.^9^ Inositol is an important metabolite which has a profound effect on gene expression and is essential for cell signaling and biological processes.^10^ Therefore, IMPA1 may affect the expression of cell‐cycle signaling associated target genes by regulating inositol metabolism. It is an interesting issue, and further studies need to be done in detail to explore the underlying mechanisms in the near future.

In addition, the gene set including those five targets and IMPA1 was significantly enriched in mTOR pathway which is important for cancer progression. Our findings also confirmed that knockdown of IMPA1 inhibits mTOR pathway in TNBC cells. In addition, these genes have also been reported to be closely related to EMT process. FAM83D was reported to regulate the EMT process of esophageal cancer cells through the Akt/GSK‐3β/signaling pathway.^20^ Additionally, FAM83D can also regulate EMT through Akt/mTOR and promote the development of lung and breast cancer.^21^, ^31^ In colorectal cancer, KIF20B mediates the EMT process through glioma‐associated oncogene 1.^22^ Overexpression of CENPF promotes the EMT process of liver cancer cells.^28^ ASPM promotes the EMT process through the PI3K/AKT pathway.^19^ Therefore, IMPA1 participates in mTOR pathway and promotes EMT process, at least in part, dependent on these target genes. Together, these findings established that IMPA1 increases TNBC progression through, at least in part, upregulating those five downstream targets and their associated mTOR pathway and EMT process in TNBC cells (Figure 7).

In summary, we found that IMPA1 was highly expressed in TNBC tissues, and exhibited oncogenic roles through mTOR pathway and EMT process. Previous studies identified lithium as a treatment to treat bipolar disorder which can cause IMPA1 KO features in mRNA level.^32^ Therefore, these data suggest that lithium supplements addition may provide an attractive strategy for improving the therapeutic response of IMPA1‐high TNBC tumors.

## AUTHORS' CONTRIBUTIONS

Shao‐Ying Yang, Yi‐Fan Xie, Tai‐Mei Zhang, Ling Deng, Li Liao, Shu‐Yuan Hu, and Yin‐Ling Zhang performed experiments and analyzed data. Fang‐Lin Zhang and DQL supervised the entire project. Shao‐Ying Yang, Fang‐Lin Zhang and Da‐Qiang Li wrote the paper with the input from all authors. All authors substantially contributed to this work, read, and approved the submitted version of this manuscript.

## FUNDING INFORMATION

The work in the Li laboratory is supported, in whole or in part, by the National Natural Science Foundation of China (82072918) to FLZ, and by the National Natural Science Foundation of China (81572584, 81772805, and 82173275) and the National Key R&D Program of China (2017YFC0908400 and 2018YFE0201600) to DQL.

## COMPETING INTERESTS

The authors declare that they have no competing interests.

## ETHICS APPROVAL AND CONSENT TO PARTICIPATE

All procedures for animal experiment were in accordance with institutional guidelines for the Care and Use of Laboratory Animals and were approved by the Animal Experiments Committee of Fudan University.

## Supporting information


Figure S1

Figure S2

Figure S3

Figure S4

Figure S5

Table S1

Table S2

Table S3

Table S4

Table S5

## Data Availability

The data used to support the findings of this study are available from the corresponding author upon request.
