# Supplementary figures and images for: Inositol monophosphatase 1 (IMPA1) promotes triple‐negative breast cancer progression through regulating mTOR pathway and EMT process
Source: Cancer Med. 2022 Jul 7;12(2):1602–15. doi: 10.1002/cam4.4970 (PMC9883559; doi:10.1002/cam4.4970)

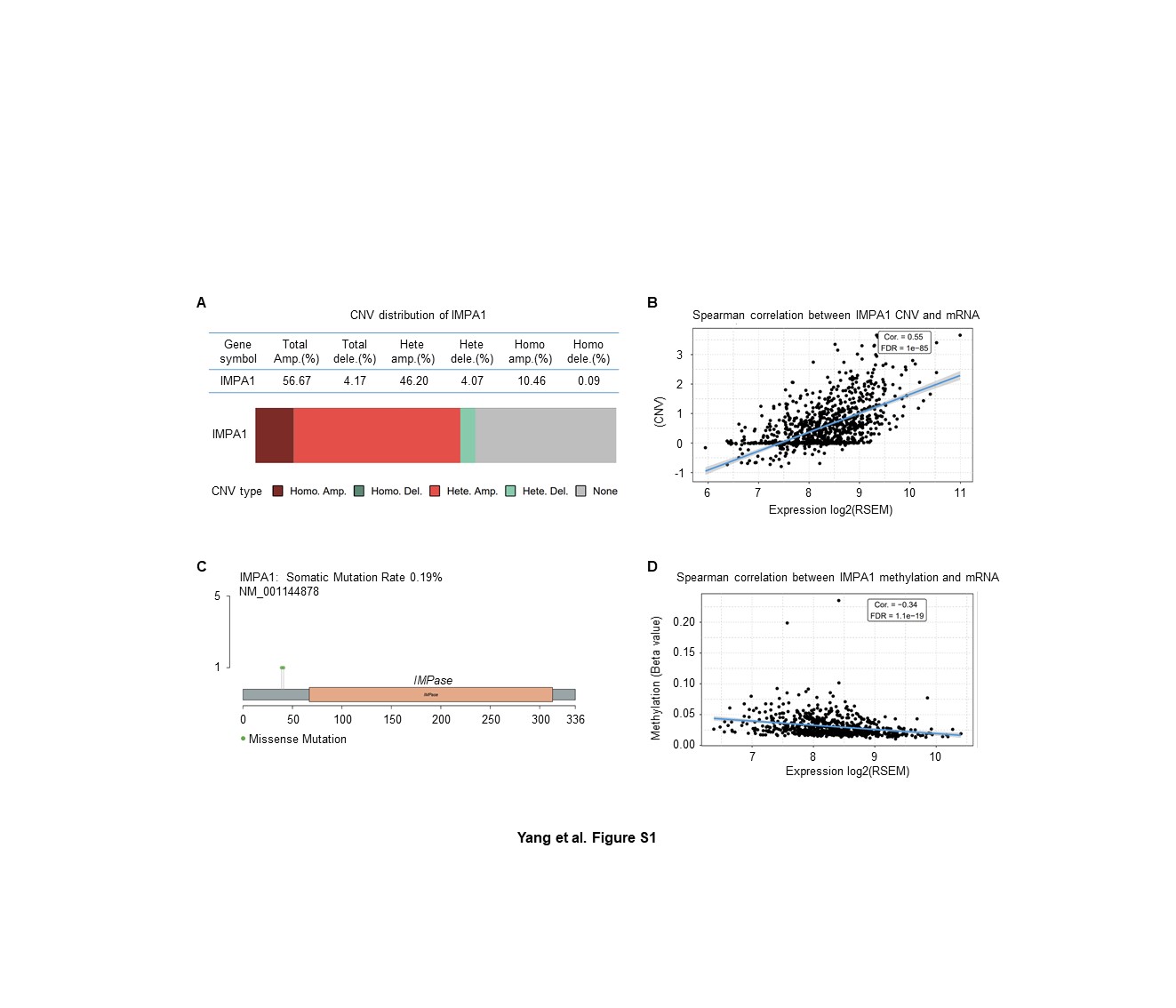

Supplement: Supplementary file 1 — Figure S1 [file CAM4-12-1602-s005.JPG]

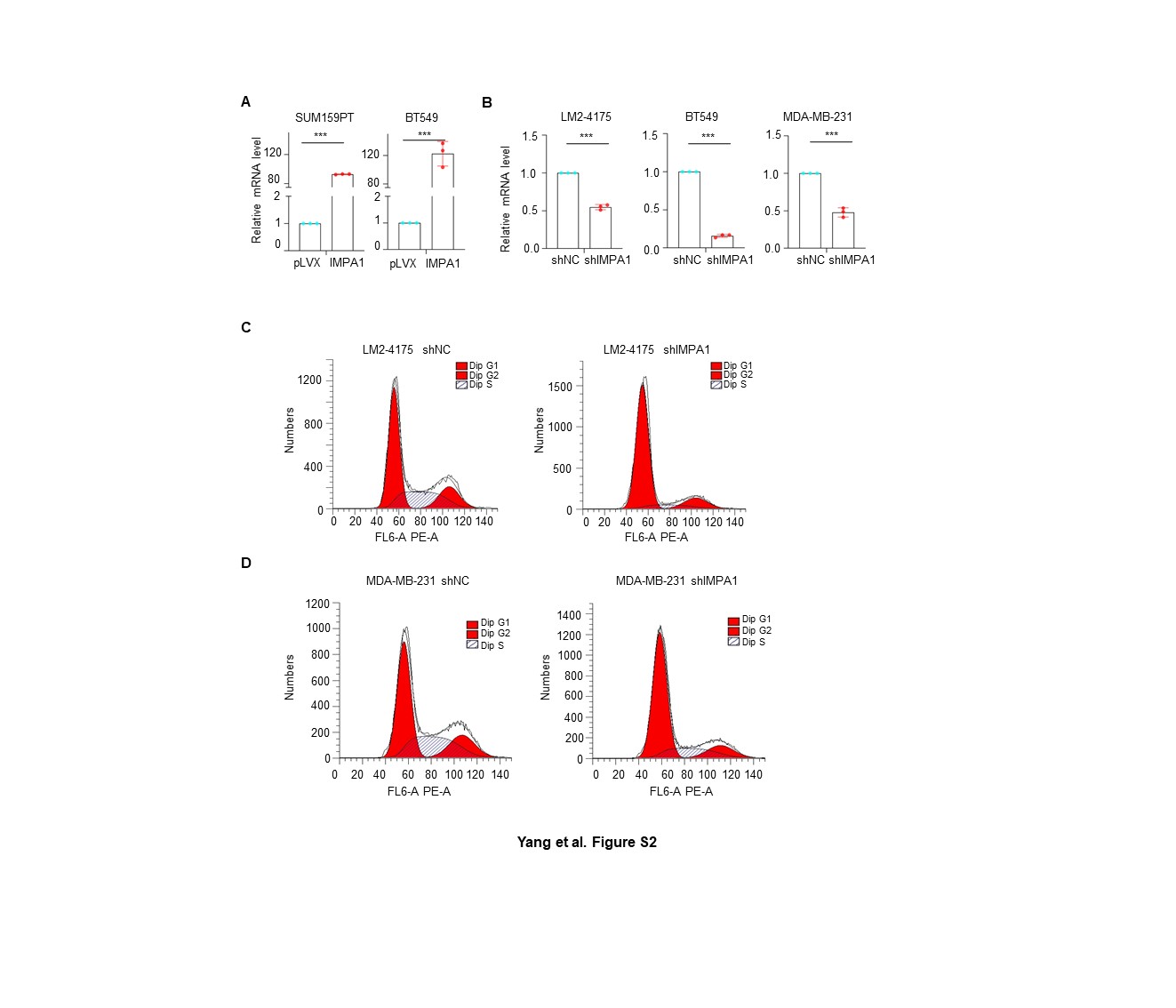

Supplement: Supplementary file 2 — Figure S2 [file CAM4-12-1602-s006.JPG]

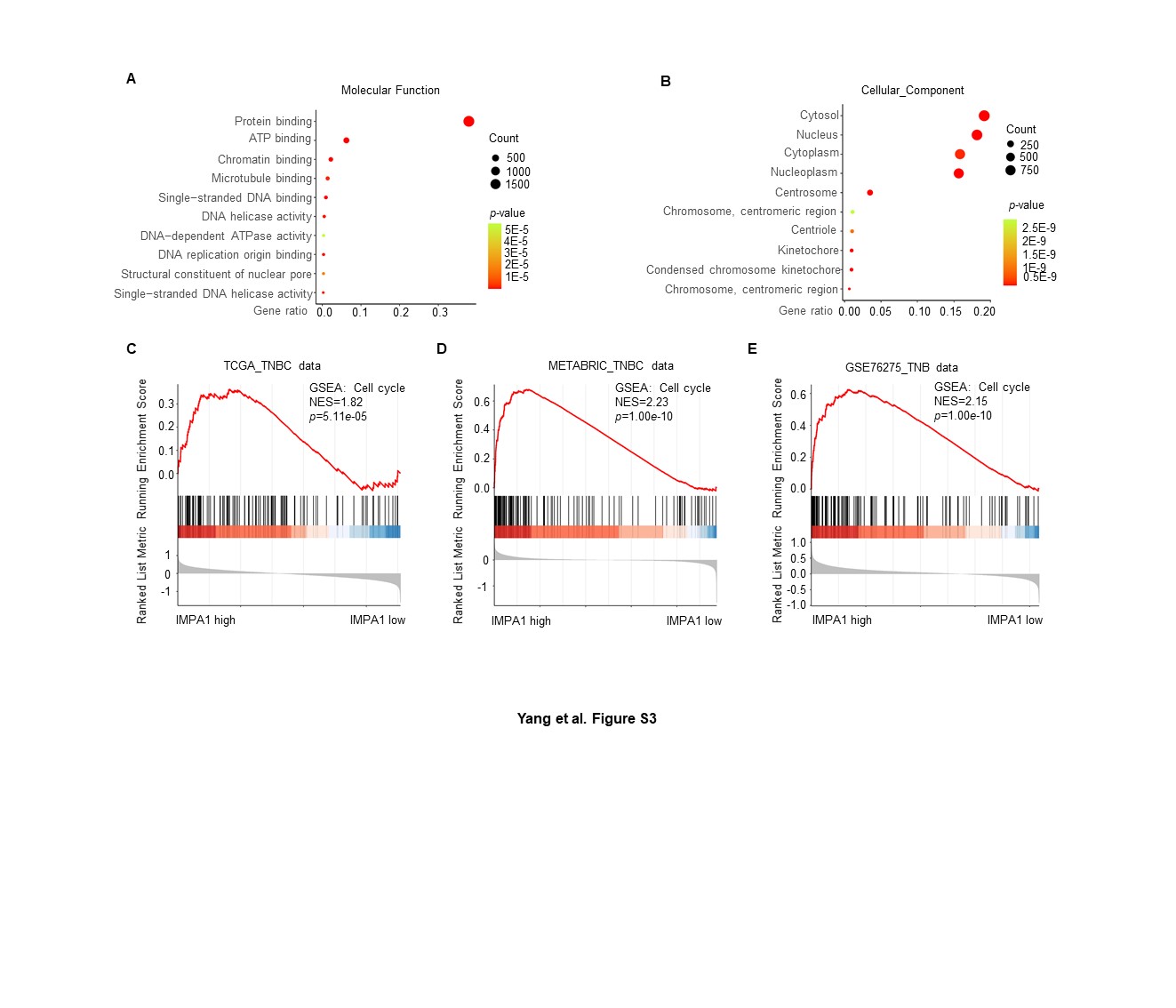

Supplement: Supplementary file 3 — Figure S3 [file CAM4-12-1602-s009.JPG]

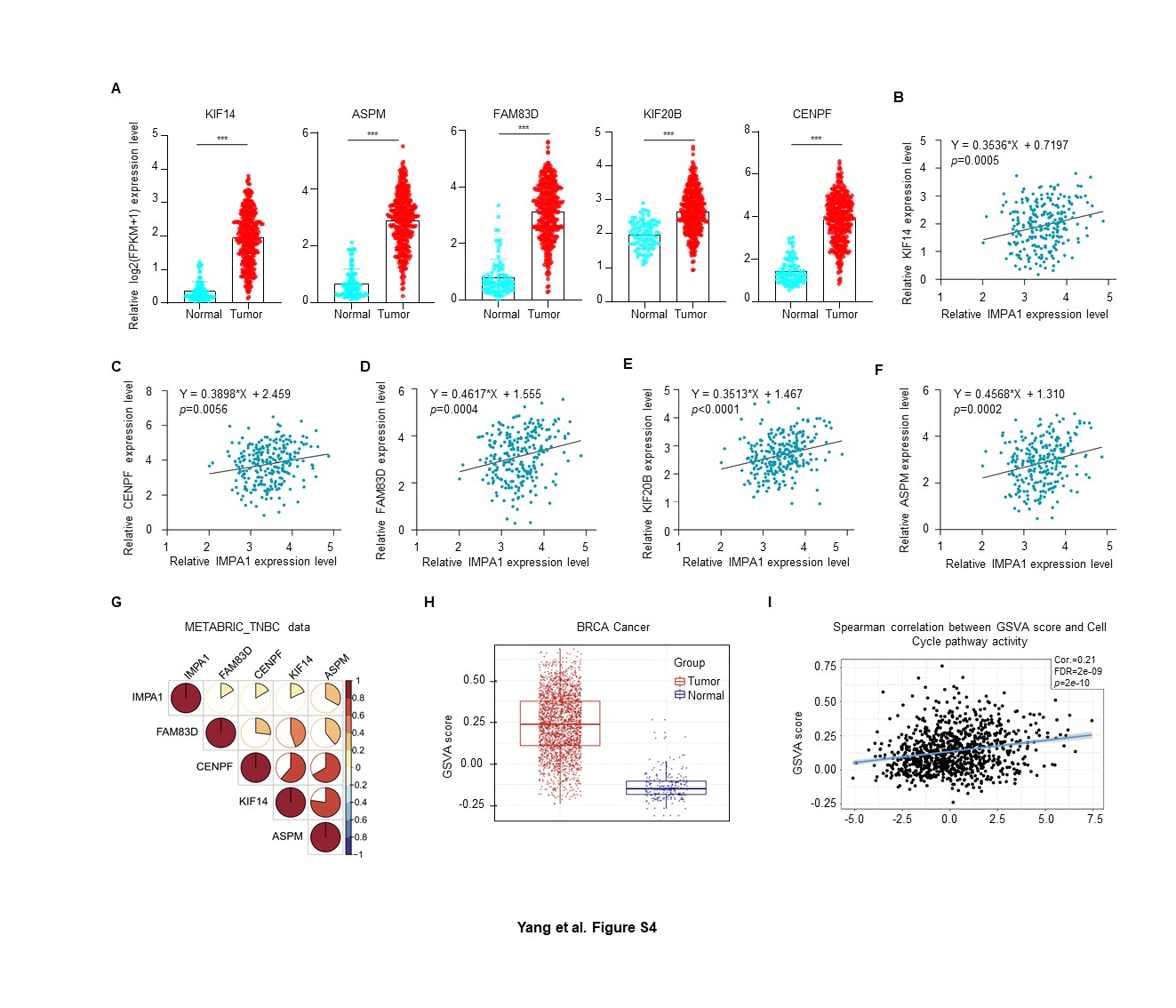

Supplement: Supplementary file 4 — Figure S4 [file CAM4-12-1602-s007.JPG]

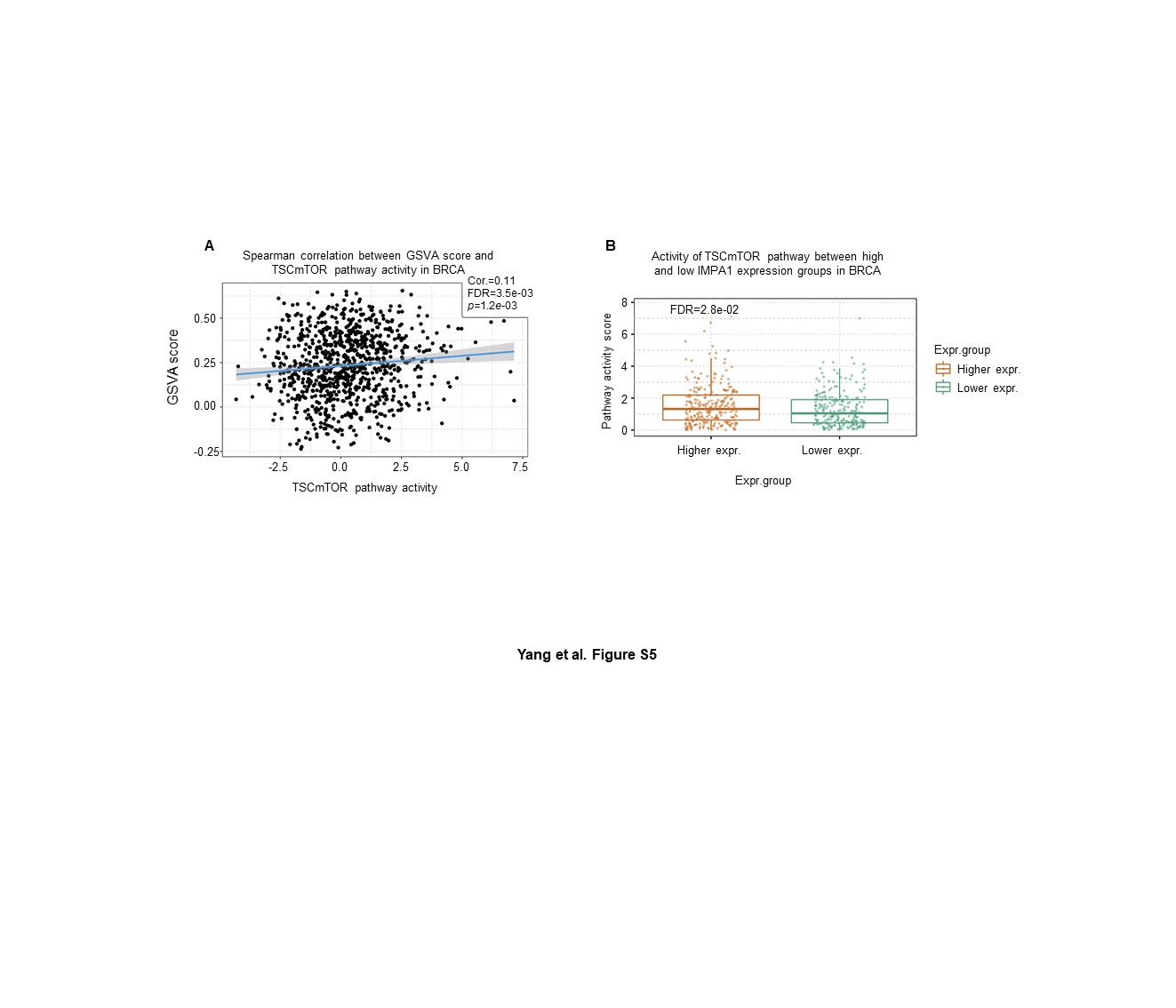

Supplement: Supplementary file 5 — Figure S5 [file CAM4-12-1602-s003.JPG]

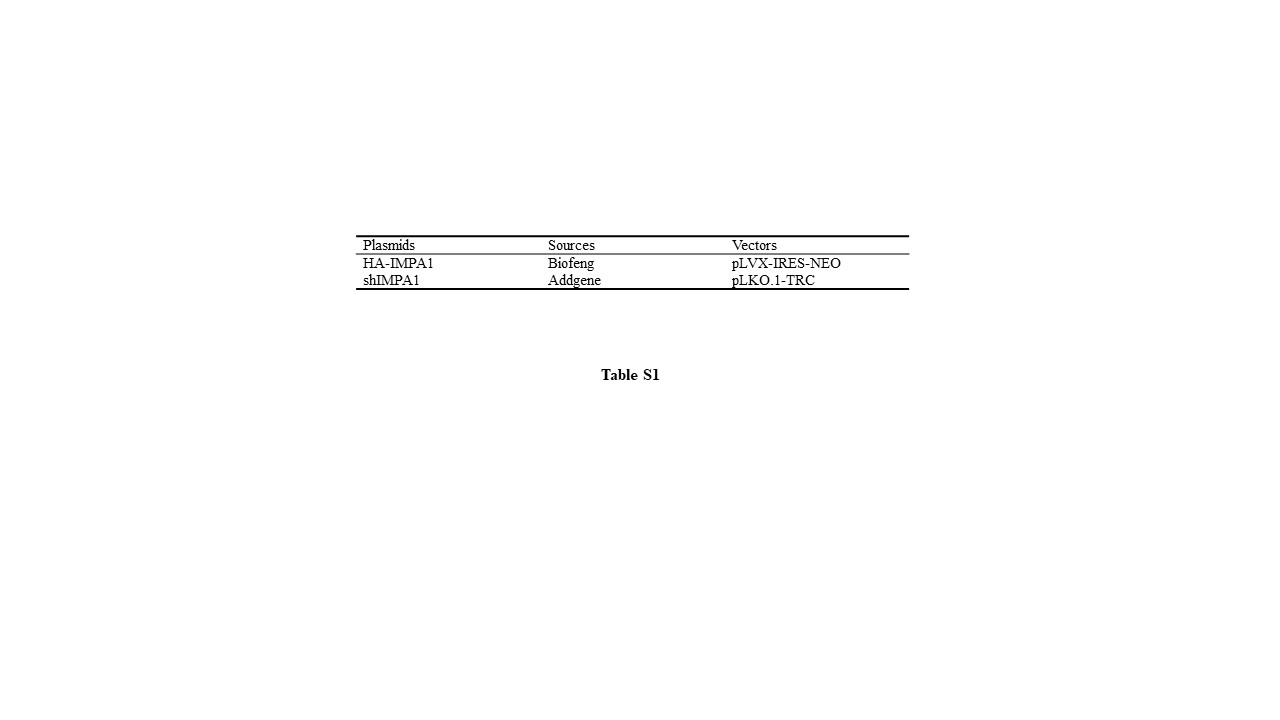

Supplement: Supplementary file 6 — Table S1 [file CAM4-12-1602-s008.JPG]

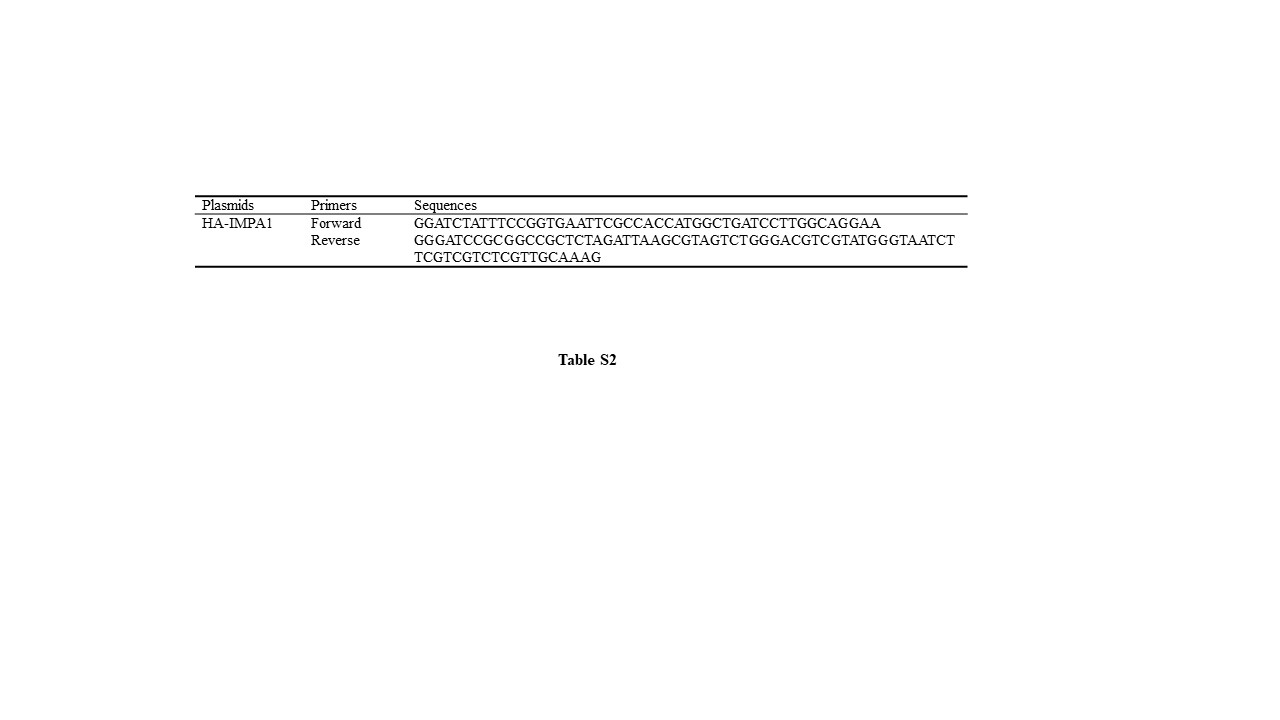

Supplement: Supplementary file 7 — Table S2 [file CAM4-12-1602-s004.JPG]

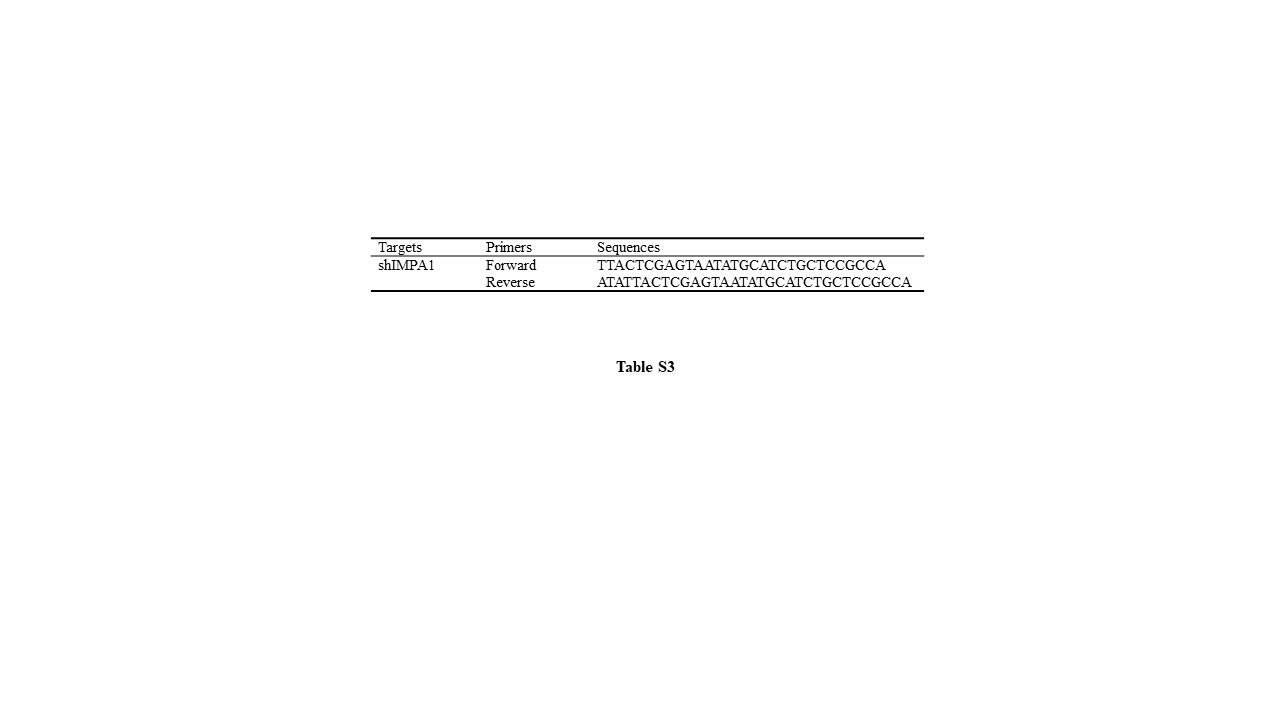

Supplement: Supplementary file 8 — Table S3 [file CAM4-12-1602-s002.JPG]

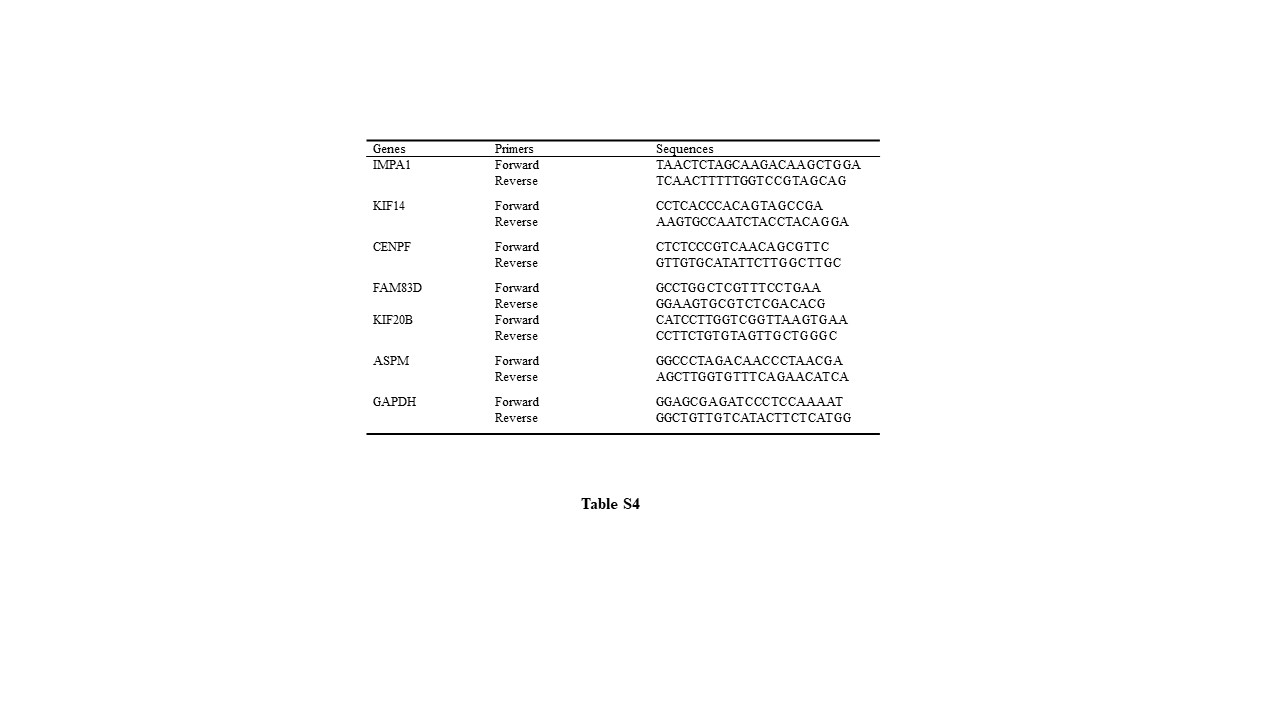

Supplement: Supplementary file 9 — Table S4 [file CAM4-12-1602-s010.JPG]

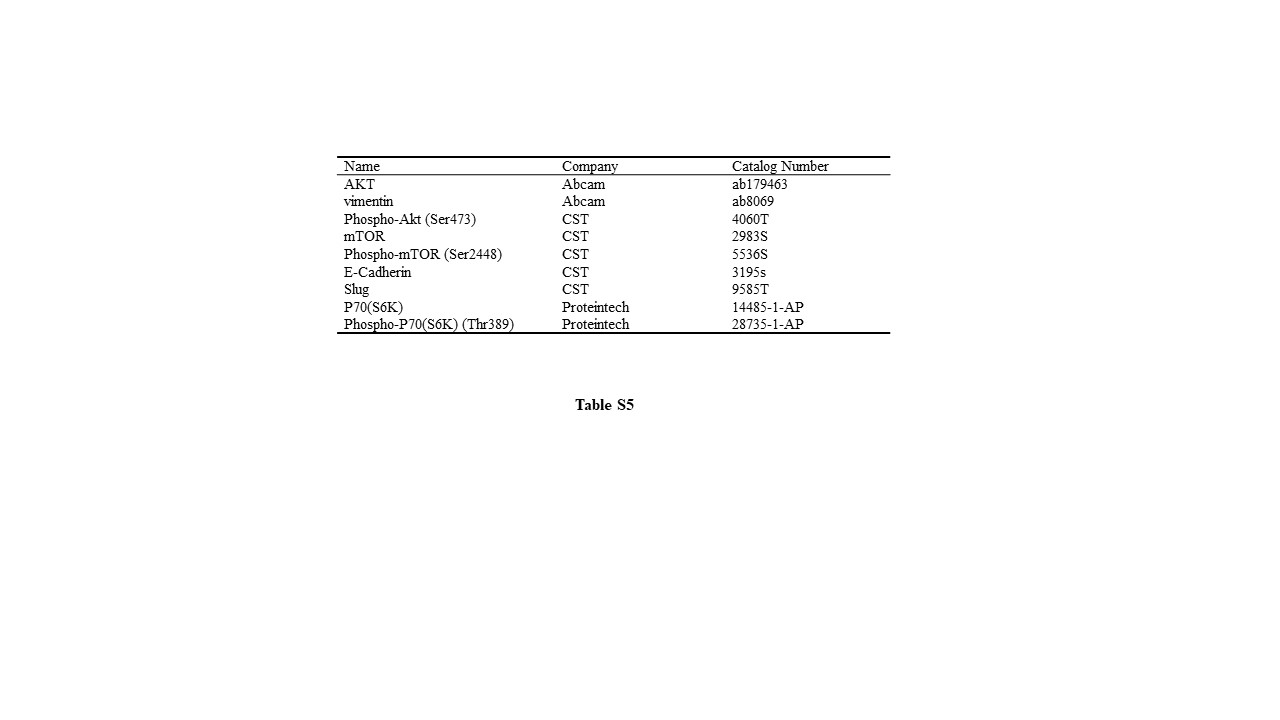

Supplement: Supplementary file 10 — Table S5 [file CAM4-12-1602-s001.JPG]
